# Downregulated Caveolin-1 expression in circulating monocytes may contribute to the pathogenesis of psoriasis

**DOI:** 10.1038/s41598-018-36767-5

**Published:** 2019-01-15

**Authors:** Naoko Takamura, Yukie Yamaguchi, Yuko Watanabe, Miho Asami, Noriko Komitsu, Michiko Aihara

**Affiliations:** Department of Environmental Immuno-Dermatology, Yokohama City University Graduate School of Medicine, Yokohama, Japan

## Abstract

Caveolin-1 (CAV-1) is the principal component of caveolae that regulates a variety of signaling molecules and receptors. Our previous study revealed CAV-1 reduction in the epidermis of patients with psoriasis, which leads to enhanced Janus kinase/signal transducer and activator of transcription activation and cytokine production, suggesting that aberrant CAV-1 expression may contribute to psoriatic inflammation. This study aimed to investigate whether abnormal modulation of CAV-1 on immune cells is involved in the pathogenesis of psoriasis. We observed that CAV-1 level in psoriasis patients was apparently reduced in peripheral blood mononuclear cells (PBMCs) and it was prominent in CD14^+^ monocytes. CAV-1 silencing in monocytes represented elevated levels of interleukin (IL)-1β and IL-6, and those had enhanced chemotaxis activity. In a murine model of psoriasis-like inflammation induced by imiquimod, we observed a significant CAV-1 reduction in PBMCs. Systemic administration of CAV-1 scaffolding domain peptide significantly improved the skin phenotype with less macrophage infiltration. Taken together, aberrant CAV-1 expression in monocytes may be involved in the pathogenesis of psoriasis.

## Introduction

Psoriasis vulgaris is a chronic immune-mediated inflammatory skin disease characterized by scaly papulosquamous plaque lesions^1^. The pathogenesis of psoriatic tissue hyperplasia is speculated to be driven by the interactions of various immune cells including macrophages, dendritic cells, and pathogenic and resident memory T cells, with enhanced representation of the interleukin (IL)-23–T helper type 17 (Th17)/ T helper type 22 (Th22) and IL-12– interferon (IFN)-γ/tumour necrosis factor (TNF)-α pathways^2^.

Caveolin-1 (CAV-1) is a 22-kDa membrane protein essential for the formation of caveolae, which serves as a signaling platform for processes such as lipid metabolism, endocytosis, and signal transduction. CAV-1 downregulates signaling molecules and receptors, which interact with the CAV-1 scaffolding domain (CSD) corresponding to amino acids 82–101 of CAV-1. We previously reported a significant reduction of CAV-1 in the epidermis of patients with psoriasis. A reduction of CAV-1 induces activation of the Janus kinase (JAK)/signal transducer and activator of transcription (STAT) pathway in psoriatic inflammation. This activation leads to further keratinocyte proliferation and cytokine/chemokine production^3^. Furthermore, we observed significant CAV-1 reduction within the epidermal hyperplasia of imiquimod (IMQ)-induced murine model of psoriasis-like skin inflammation and restoration of CAV-1 function in this mice improved skin phenotype.

CAV-1 is also identified in various immune cells, including monocytes/macrophages, polymorphonuclear cells (PMNs), mast cells, and lymphocytes^4–7^. Recently, CAV-1 has been implicated as a modulator of innate immunity and inflammation. CAV-1 inhibits the expression of pro-inflammatory cytokines from macrophages by regulating the activation of mitogen-activated protein kinase (MAPK) family members^8^. Some studies reported that reduction of CAV-1 in immune cells is implicated in various human diseases, fibrotic lung diseases, graft versus host disease, atherosclerosis, and diabetic neuropathy^9–12^. In this study, we hypothesized that abnormal CAV-1 level not only in keratinocytes but also in leukocytes may be involved in the pathogenesis of psoriasis. Therefore, we evaluated CAV-1 levels in leukocytes of patients with psoriasis and investigated their roles in the pathogenesis of psoriasis.

## Results

### CAV-1 is downregulated in PBMCs and PMNs of patients with psoriasis

**Figure 1:**
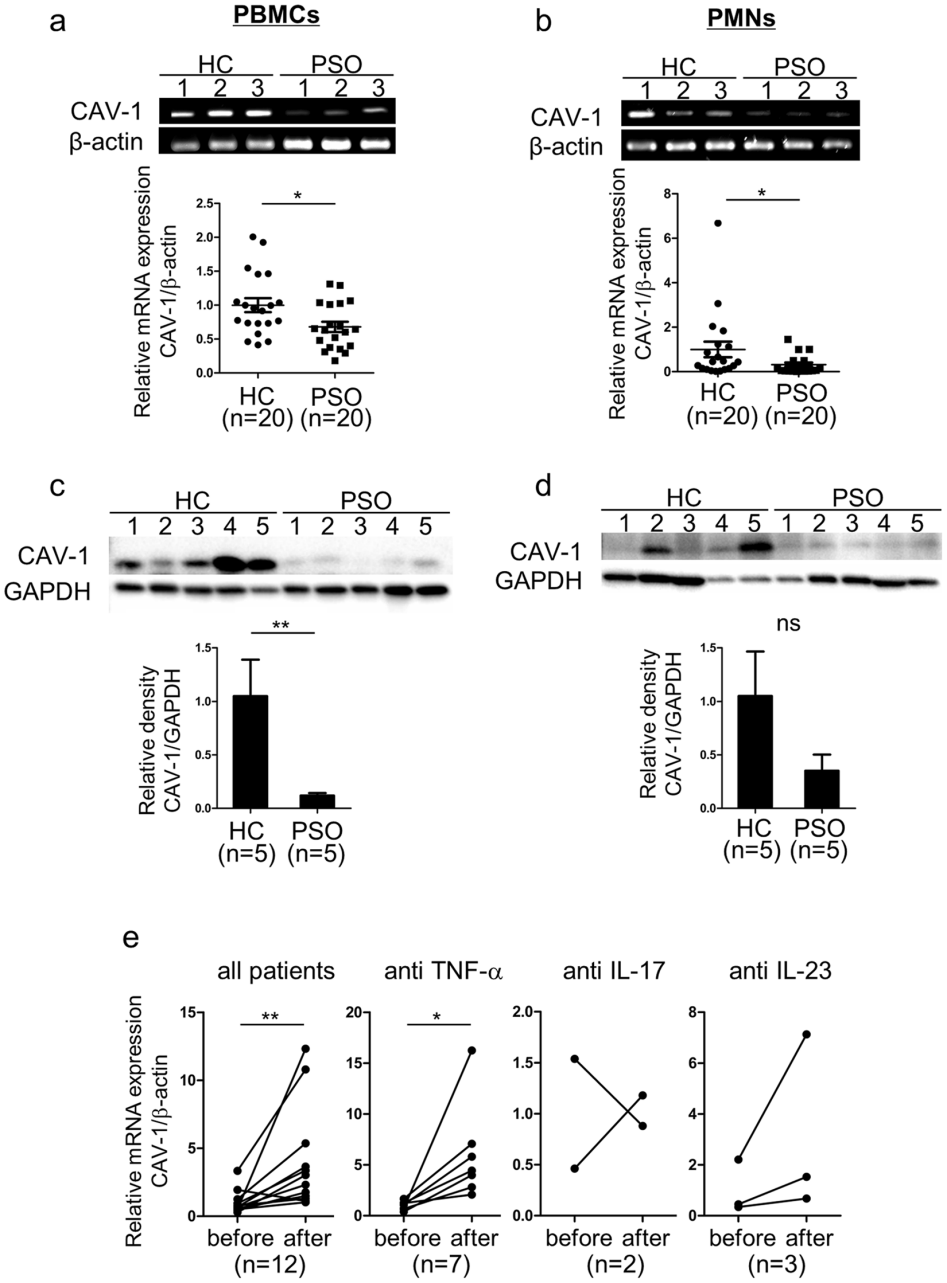
Decreased CAV-1 expression in PBMCs and PMNs from patients with psoriasis. CAV-1 expression in PBMCs (**a**,**c**) and PMNs (**b**,**d**) of patients with psoriasis (PSO) and healthy subjects (HC) was evaluated using semi-quantitative and qPCR (**a**,**b**), and immunoblotting (**c**,**d**). (**a**,**b**) Representative semi-quantitative PCR images from three patients and healthy controls are shown. Twenty individual samples were subjected to qPCR in duplicate. Mean of normalized gene expression levels in HC was arbitrarily set at 1. Mann-Whitney U test, *P < 0.05. (**c**,**d**) Representative images of immunoblotting from five patients and healthy subjects are shown. CAV-1 protein levels were quantified using densitometry and expressed as the ratio of CAV-1 to GAPDH. Mean of normalized density level in HC was arbitrarily set at 1. Unpaired-t-test, **P < 0.01; ns: not significant. (**e**) Graphs represent changes of CAV-1 expressions in PBMCs of patients with psoriasis before and after the treatment with biologics. Wilcoxon test, ***P < 0.001, ns: not significant. All data are shown as mean ± SD. Blots shown are derived from multiple gels. The gels were run under the same experimental conditions. The membrane was cut based on molecular weight. All full-length blots/gels are presented in Supplementary Fig. S3.

We first evaluated CAV-1 levels in leukocytes of patients with psoriasis. mRNA expression and protein level of CAV-1 were determined using quantitative polymerase chain reaction (qPCR) and immunoblotting in peripheral blood mononuclear cells (PBMCs) and PMNs. PBMCs and PMNs were isolated from patients with psoriasis vulgaris, who had no history of biologic use and no current use of immunosuppressant, and from healthy subjects. As shown in Fig. 1, CAV-1 mRNA expression in PBMCs (Fig. 1a) and PMNs (Fig. 1b) of patients with psoriasis was significantly reduced compared with that of healthy subjects (P = 0.022, P = 0.047, respectively). Similarly, CAV-1 protein level in PBMCs of patients with psoriasis was significantly decreased compared with that of controls (Fig. 1c, P = 0.025). In PMNs, CAV-1 protein levels varied in healthy subjects. We found a trend towards reduced CAV-1 protein level in the PMNs of patients with psoriasis, but the difference did not reach statistical significance (Fig. 1d, P = 0.151). Therefore, we focused more on PBMCs and conducted further experiments. In some patients with psoriasis who were treated with biologics, CAV-1 expression in PBMCs was serially analysed before and after treatments. Interestingly, recovery of CAV-1 level in PBMCs was observed as clinical improvement by treatments (Fig. 1e, P = 0.002 in total, P = 0.016 in patients treated with TNF-α inhibitors).

### CAV-1 reduction is predominant in CD14^+^ monocytes

**Figure 2:**
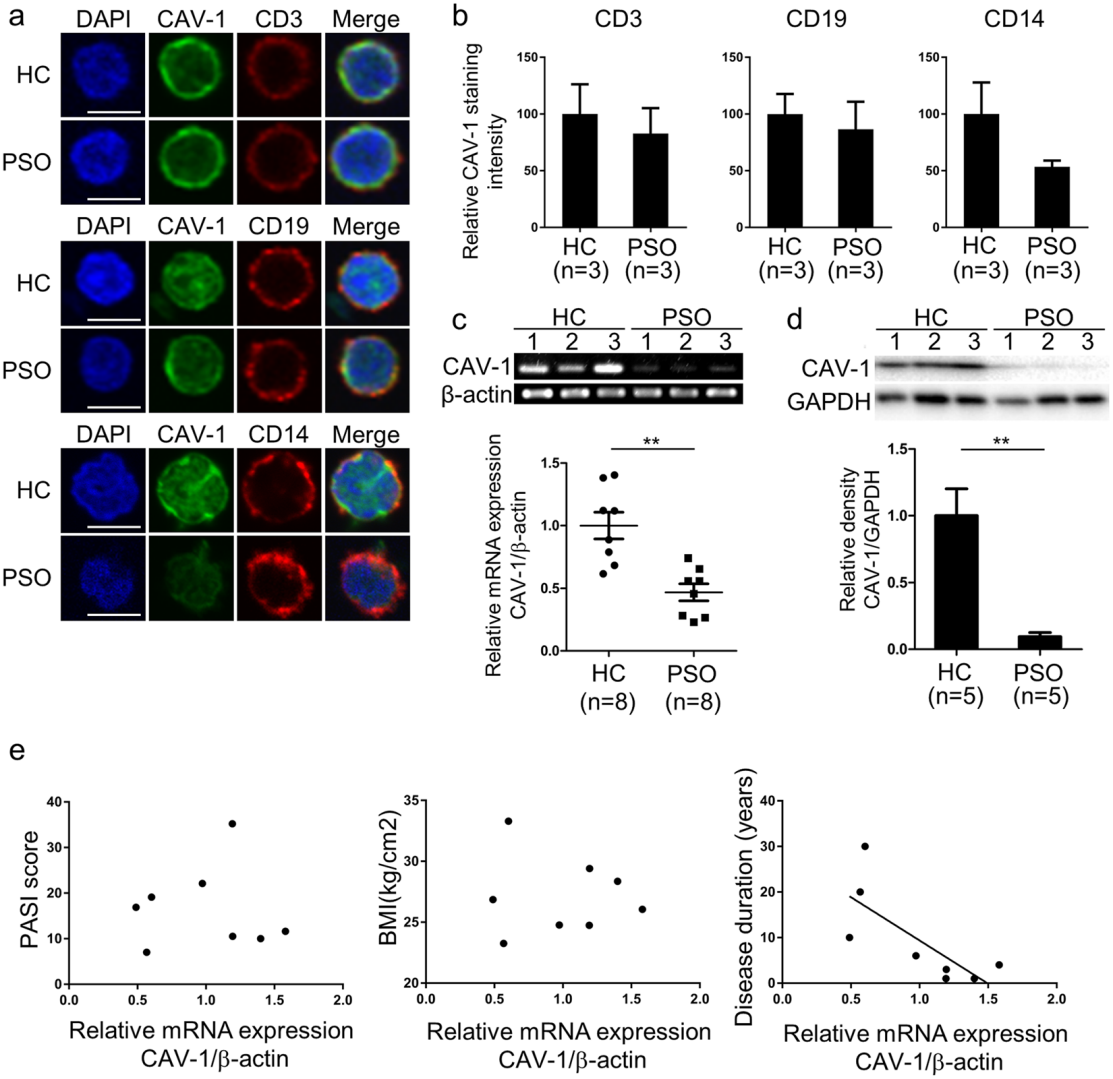
Diminished CAV-1 expression in circulating monocytes of patient with psoriasis. (**a**,**b**) Immunofluorescent intensities of CAV-1 in circulating T cells, B cells, and monocytes were determined. (**a**) PBMCs isolated from psoriasis patients (PSO) and healthy subjects (HC) were stained with CAV-1 (green) and with either CD3, CD19, or CD14 (red). DAPI was used to identify nuclei. Scale Bars = 5 μm. (**b**) CAV-1 intensities in randomly chosen CD3^+^ T cells, CD19^+^ B cells, and CD14^+^ monocytes were quantified. The data were obtained from 20 cells of each three patients and healthy subjects. Mean intensity level in HC was arbitrarily set at 100. (**c**,**d**) CAV-1 levels in purified monocytes were evaluated using semi-quantitative and qPCR (**c**), and immunoblotting (**d**). (**c**) Representative semi-quantitative PCR images from three patients and healthy controls are shown. Eight individual samples were subjected to qPCR in duplicate. Mean of normalized gene expression level in HC was arbitrarily set at 1. Mann-Whitney U test, **P < 0.01. (**d**) Representative images of immunoblotting from three patients and healthy subjects are shown. CAV-1 protein levels were quantified using densitometry and expressed as the ratio of CAV-1 to GAPDH (n = 5). Mean of normalized density level in HC was arbitrarily set at 1. Unpaired-t test, **P < 0.01. All data are shown as mean ± SD. Blots shown are derived from multiple gels. The gels were run under the same experimental conditions. The membrane was cut based on molecular weight. All full-length blots/gels are presented in Supplementary Fig. S4. (**e**) Correlations between CAV-1 mRNA expression level in monocytes of psoriasis patients and clinical features: PASI score, BMI, and disease duration (n = 8).

To evaluate which type of cells was important in CAV-1 downregulation, immunofluorescent staining of PBMCs was performed and CAV-1 intensities quantified by fluorescent microscopy were compared between patients with psoriasis and controls in CD3^+^ T cells, CD19^+^ B cells, and CD14^+^ monocytes. As shown in Fig. 2a,b, the levels of CAV-1 intensity in each type of cells in patients with psoriasis were lower than those in controls (reduction rate: 17 ± 10%, 16 ± 20%, and 43 ± 14% in T cells, B cells, and monocytes, respectively). Since CAV-1 reduction was most prominent in circulating monocytes, we confirmed that CAV-1 mRNA and protein levels in monocytes were purified from PBMCs of patients with psoriasis in comparison with those of healthy subjects by qPCR and immunoblotting. CAV-1 levels in monocytes of patients with psoriasis were significantly decreased compared with those of controls (Fig. 2c,d, P = 0.001, P = 0.002, respectively). To evaluate if CAV-1 levels in monocytes directly represent clinical parameters in psoriasis patients, we analysed correlations between CAV-1 expression levels in monocytes and clinical features, such as Psoriasis Area and Severity Index (PASI) score, body mass index (BMI), and disease duration in psoriasis patients. As shown in Fig. 2e, there were no correlations between monocyte CAV-1 levels and PASI score or BMI, but CAV-1 levels negatively correlated with disease duration (r = −0.011, P = 0.98, r = −0.078, P = 0.855, r = −0.747, P = 0.033, respectively, Fig. 2e).

### Enhanced levels of inflammatory cytokines in CAV-1-silenced monocytes

**Figure 3:**
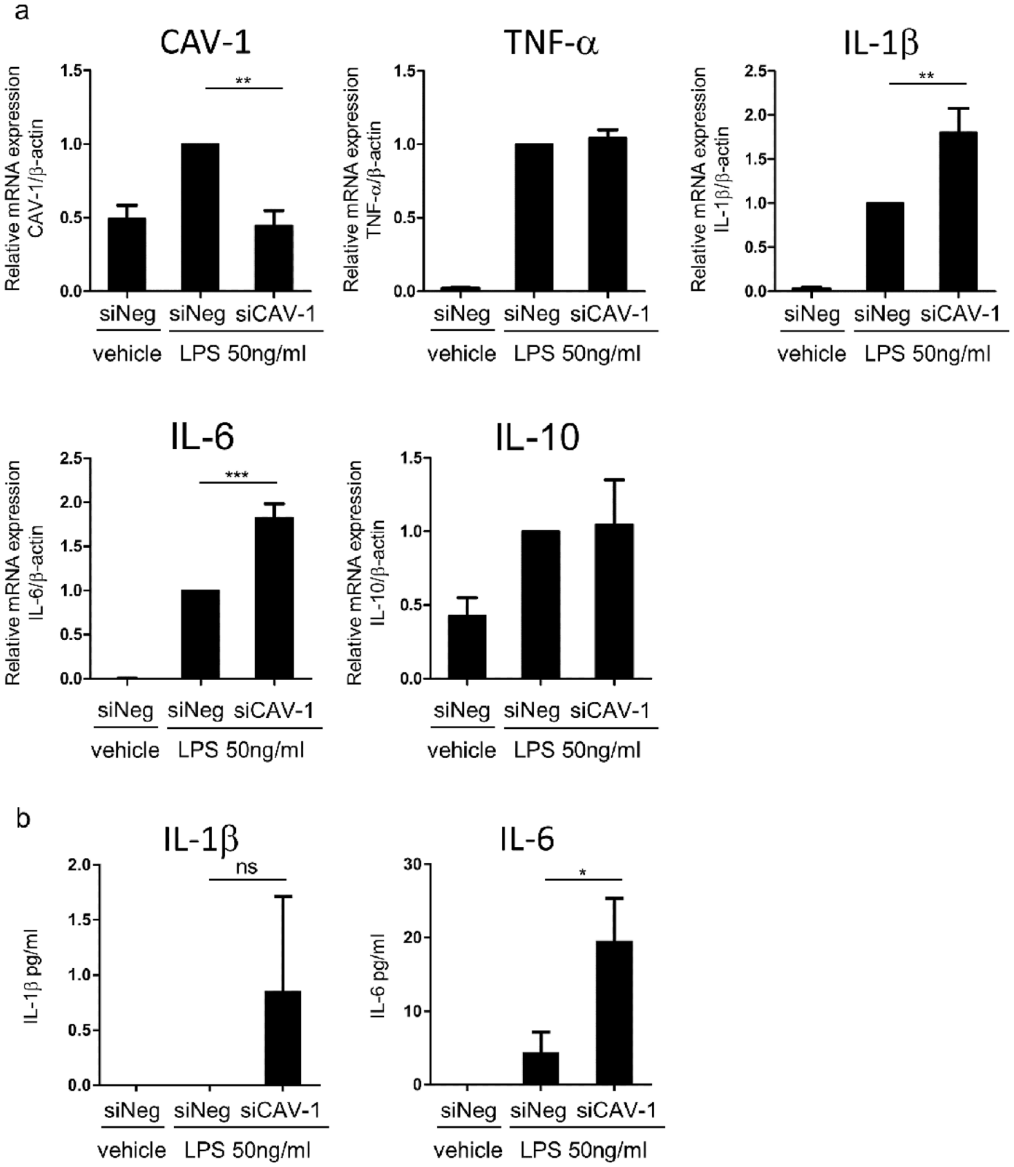
Enhanced cytokine production in CAV-1-silenced monocytes. The relative gene expression and released cytokine levels were determined in CAV-1-silenced (siCAV-1) and control siRNA (siNeg) monocytes. Monocytes were obtained from three donors and stimulated with LPS or PBS (Vehicle) for 4 hours. The experiments were performed on three independent experiments. (**a**) The relative gene expression levels of CAV-1, TNF-α, IL-1β, IL-6, and IL-10 were determined by quantitative PCR. Normalized gene expression levels in siNeg Monocytes with LPS stimulation alone were arbitrarily set at 1. Graph indicates the mean ± SD. One-way analysis of variance (post hoc Tukey), *P < 0.05; **P < 0.01; ***P < 0.001. (b) IL-1β and IL-6 levels in supernatants of those monocytes were determined using an enzyme-linked immunosorbent assay. Graph indicates the mean ± SD. One-way analysis of variance (post hoc Dunnett), *P < 0.05; ns, not significant.

In our previous findings, reduced CAV-1 level in psoriatic keratinocytes enhances the activation of several signaling molecules and increases cytokine/chemokine productions^3^. Therefore, we hypothesized that CAV-1 reduction in monocytes may also influence the pathogenesis of psoriasis by modulating cytokine production. CAV-1 was silenced in primary human monocytes by RNA interference, and gene expression levels of psoriasis-related cytokines induced by lipopolysaccharide (LPS) were evaluated using qPCR. As shown in Fig. 3, the mRNA expression levels of IL-1β and IL-6 in CAV-1-silenced monocytes were significantly increased compared with those in control monocytes (Fig. 3a, P = 0.003, P = 0.0002, respectively). However, no differences were observed in the levels of TNF-α and IL-10, and IL-17A expression was undetected (data not shown). We also measured levels of IL-1β and IL-6 released into the supernatants. The levels of IL-6 in supernatants of CAV-1-silenced monocyte were significantly increased compared with those in controls (Fig. 3b, P = 0.045), although those IL-1β levels were found to be increased, but it did not reach statistically significant (Fig. 3b, P = 0.418).

### Increased migration activity in CAV-1-silenced monocytes

**Figure 4:**
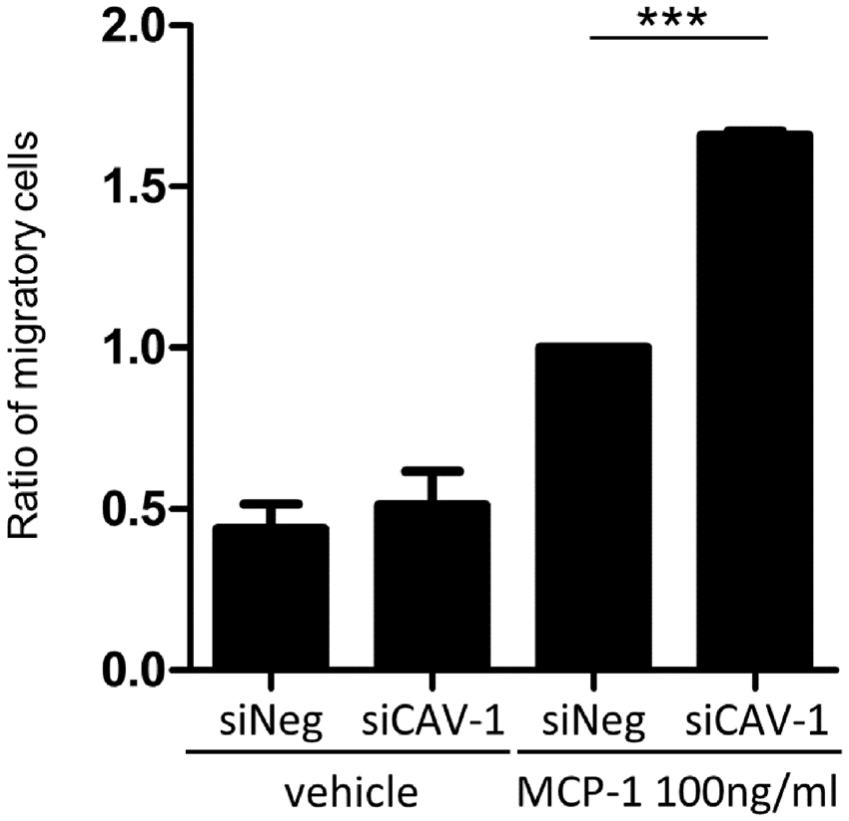
Enhanced chemoattractant activity in CAV-1-silenced monocytes. The chemoattractant activity of monocytes toward MCP-1 was assessed by chemotaxis assay. Cav-1-silenced (siCAV-1) or control monocytes (siNeg) were seeded in the upper compartment, and the medium containing 100 ng/ml MCP-1 was then added to the lower compartment. After a 90-minute incubation, cells were collected from both compartments and manually counted. Graph displays the ratio of migrating monocytes. Monocytes were obtained from three donors, and results represented three independent experiments. Data are shown as a mean ± SD. One-way analysis of variance (post hoc Tukey), ***P < 0.001 (siNeg + MCP-1 vs. siCAV-1 + MCP-1).

Infiltration of macrophages and high monocyte chemotactic protein (MCP)-1 expression level were found in psoriasis lesional skin^13,14^. Hence, we evaluated whether a reduction of CAV-1 is involved in monocyte infiltration to dermis. Chemotaxis assay using CAV-1 silenced monocytes toward MCP-1 was performed. The ratio of migrated cells in CAV-1-silenced monocytes was significantly higher than that in control monocytes (Fig. 4, P = 0.0005). Taken these results together, downregulated CAV-1 in monocytes may have pathophysiological function, e.g., exacerbated migration and enhanced pro-inflammatory cytokine production.

### Systemic administration of CSD peptide improves the skin phenotype in IMQ-induced murine model of psoriasis-like skin inflammation

**Figure 5:**
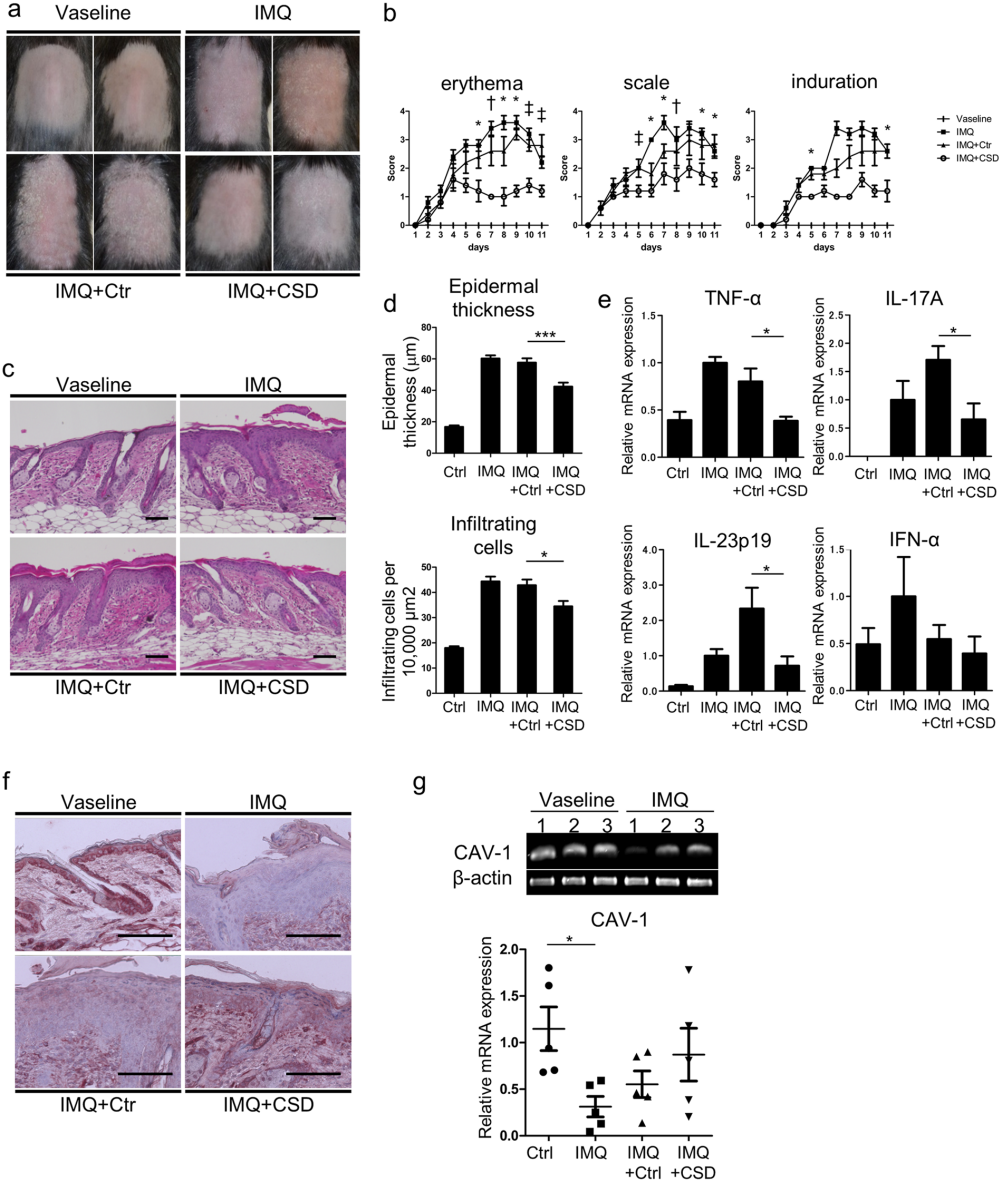
Phenotype of IMQ-induced murine model of psoriasis-like skin inflammation and suppressed inflammation by systemic administration of CSD peptide. (**a**) Mice were treated with IMQ alone (IMQ), IMQ and the CAV-1 scaffolding domain (CSD) peptide (IMQ + CSD), IMQ and the control peptide (IMQ + Ctr), or Vaseline for 10 days. Representative images of mice are shown. (**b**) Skin scores represent the erythema, induration, and scale scores in each group: Vaseline (lines); IMQ alone (squares); IMQ + CSD (circles); and IMQ + Ctr (triangles). Graphs indicate the mean ± SD of each group (n = 6). One-way analysis of variance (post hoc Tukey), *P < 0.05; ^†^P < 0.01; ^‡^P < 0.001 (IMQ vs. IMQ + CSD). (**c**) Representative haematoxylin and eosin staining of mice skin. Scale bar = 100 μm. (**d**) The epidermal thickness and the number of infiltrating cells were analysed. Graphs indicate the mean ± SD of each group (n = 6). One-way analysis of variance (post hoc Tukey), *P < 0.05, ***P < 0.001 (IMQ + Ctr vs. IMQ + CSD). (**e**) Relative mRNA expression levels of TNF-α, IL-17A, IL-23p19, and IFN-α in the dermis were determined by qPCR in duplicate. Graphs indicate the mean ± SD of each group (n = 5). One-way analysis of variance (post hoc Tukey), *P < 0.05 (IMQ + Ctr vs. IMQ + CSD). (**f**) Immunohistochemical analysis of CAV-1. Scale bar = 100 μm. (**g**) CAV-1 expression in PBMCs in each group was determined using semi-quantitative and qPCR. Graphs indicate the mean ± SD of each group (n = 5). One-way analysis of variance (post hoc Tukey), *P < 0.05 (Ctr vs. IMQ). Gels shown are derived from multiple gels. The gels were run under the same experimental conditions. All full-length gels are presented in Supplementary Fig. S5.

**Figure 6:**
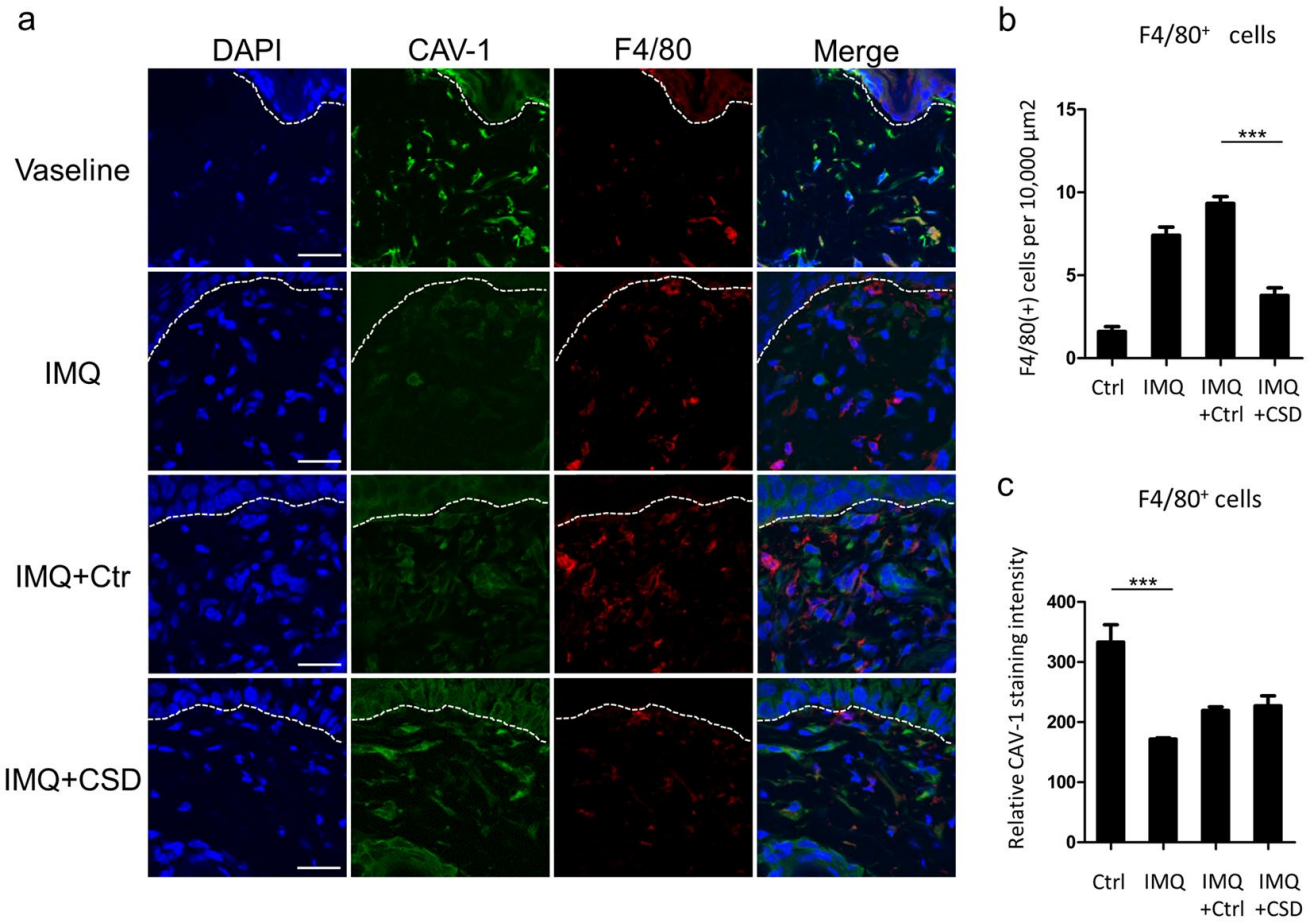
Reduced infiltration of dermal macrophages in IMQ-induced murine model of psoriasis-like skin inflammation by CSD treatment. (**a**) Representative immunofluorescent images of mice skin sections. Skin sections were stained with antibodies against CAV-1 (green) and F4/80 (red). DAPI was used to identify nuclei. Scale bar = 20 μm. (**b**) The number of infiltrating F4/80^+^ cells was analysed. The data were obtained from 5 random fields of view for each mouse. Graphs indicate the mean ± SD of each group (n = 6). One-way analysis of variance (post hoc Tukey), ***P < 0.001 (IMQ + Ctr vs. IMQ + CSD). (**c**) Intensity levels of CAV-1 in F4/80^+^ cells were evaluated by immunofluorescent analysis. The data were obtained from 10 cells from 10 random fields of view for each mouse (n = 6). Mean intensity level in mice treated with IMQ alone was arbitrarily set at 1. Graphs indicate the mean ± SD of each group (n = 6). One-way analysis of variance (post hoc Tukey), ***P < 0.001 (Vaseline Ctr vs. IMQ).

CAV-1 reduction is found in the epidermis of a murine model of psoriasis-like inflammation induced by IMQ, and intradermal injection of CSD peptide improves skin phenotype^3^. CSD peptides can cross the plasma membrane as they are synthesized by fusion to the antennapedia internalization sequence^15^. They reverse the effects of CAV-1 depletion in many systems by substituting for the full-length molecule. Since diminished CAV-1 level was observed not only in the epidermis but also in leukocytes of patients with psoriasis, we hypothesized that an aberrant expression of CAV-1 is present in leukocytes of this mouse model. We also hypothesized that systemic administration of CSD peptide may influence clinical phenotype by modulating CAV-1 level of immune cells. Intraperitoneally administered CSD peptide significantly improved skin phenotype compared with control peptide-treated mice (Fig. 5a,b). Histological analysis revealed that epidermal thickness and number of infiltrating cells were greatly reduced in the CSD mice (Fig. 5c,d). Moreover, systemic CSD treatment significantly decreased cytokine expressions, TNF-α, IL-17A, and IL-23p19, in the skin compared with the controls (Fig. 5e, P = 0.022, P = 0.04, and P = 0.018, respectively). We next evaluated CAV-1 expressions of epidermis and leukocytes in these mice. Supporting our previous study, IMQ treatment apparently reduced CAV-1 level in the epidermis as seen in the immunohistochemical analysis, and CAV-1 level was restored by systemic CSD treatment (Fig. 5f). Notably, IMQ treatment also significantly suppressed CAV-1 expression in PBMCs (P = 0.047), and its level tended to be improved by CSD administration (Fig. 5g). This result suggests that abnormality of CAV-1 level in leukocytes is involved *in vivo* in IMQ-induced psoriasis-like mouse model. To clarify the participation of monocytes/macrophages in this model, we further evaluated infiltrated dermal macrophages in association with CAV-1. As shown in Fig. 6a,b, the number of F4/80^+^ dermal macrophages was evidently increased by IMQ application. Meanwhile, CSD treatment significantly suppressed its infiltration (P < 0.0001). Furthermore, the fluorescent intensity of CAV-1 in F4/80^+^ cells was assessed using immunofluorescent microscopy. Infiltrated dermal macrophages had clearly less CAV-1 signal in IMQ-treated mice than in control mice (Fig. 6c, P = 0.0007). However, in CSD mice, CAV-1 signal in macrophages may be higher than that in IMQ mice. No significant differences were found among treatment groups. Additionally, to confirm the efficacy of systemically administrated CSD in another psoriasis-like skin inflammation, an IL-23-induced psoriasis-like murine model was used. We again observed that intraperitoneally administered CSD peptide significantly improved skin phenotype (Supplementary Fig. S1). This result suggests that CAV-1 abnormality is also involved *in vivo* in IL-23-induced psoriasis-like skin inflammation.

## Discussion

The present study is the first to demonstrate that CAV-1 expression is downregulated not only in keratinocytes but also in leukocytes/monocytes of patients with psoriasis and the murine model. It also showed that diminished CAV-1 in monocytes may modulate cell behaviour in a manner that promotes psoriasis pathology by augmenting cytokine production and migration activity.

With regard to trigger for reduced CAV-1 level, we previously proposed that psoriasis-related cytokines themselves downregulated CAV-1 expression in keratinocytes. Similarly, in monocytes and other cell types, several studies showed that CAV-1 expression is inhibited by TNF-α, transforming growth factor-β, and IL-17A in other pathologies^3,9,16^. Since such psoriasis-related pro-inflammatory cytokines were high in the serum of patients with psoriasis^17–19^, it may be reasonable that a cytokine-rich environment in psoriasis would favour CAV-1 reduction in leukocytes/monocytes. In this regard, cytokine removal by biologics in patients with psoriasis resulted in increased CAV-1 level in PBMCs with clinical improvement in this study. Interestingly, we found an inverse correlation between CAV-1 levels in monocytes and disease duration in psoriasis patients. As disease duration is closely involved in the process of prolonged systemic inflammation, CAV-1 deficiency in monocytes may directly contribute to the chronicity of psoriatic inflammation.

The pathophysiological contribution of monocytes in the pathogenesis of psoriasis is not completely elucidated, but monocytes are speculated to play pivotal roles. Monocytes from patients with psoriasis are known to overproduce cytokines (e.g., TNF-α, IL-1β, IL-6, and IL-8)^20,21^. They are highly sensitive to tiny stimuli as a small amount of LPS could induce inflammatory cytokines in comparison with healthy monocytes^22^. The recent study by Yamanaka *et al*. revealed that the percentage of CD14^high^ activated monocytes, which release high levels of TNF-α, IL-6, and IL-10, is increased in patients with severe psoriasis compared to those with moderate psoriasis and normal controls. Anti-TNF-α therapy normalizes the ratio of activated monocytes but does not affect cytokine production on lymphocytes, suggesting the importance of monocytes as a contributor to the clinical condition of psoriasis^23^. We observed that CAV-1-reduced monocytes exacerbated the production of psoriasis-related inflammatory cytokines (IL-1β and IL-6). In this regard, several lines of evidence revealed that CAV-1 interacts with various signaling molecules and negatively regulates those signaling in many types of cells and tissues, and downregulated CAV-1 directly potentiates signaling in many pathologies^3,24^. Predictably, hyperactivation of several members of the MAPK family of signaling molecules (ERK, JNK, p38) was also observed in CAV-1-reduced monocytes from scleroderma patients^9^. Therefore, the activated signaling molecules as a result of CAV-1 downregulation in monocytes are presumed to lead to overproduction of inflammatory cytokines in our study.

Infiltration of monocytes/macrophages in lesional skin is a hallmark of psoriasis. We observed that the migration activity toward MCP-1 was enhanced in CAV-1-silenced monocytes *in vitro* and CSD peptides diminished macrophage infiltration *in vivo* in a mouse model. These results partially contradict the results of a study by Yi Fu *et al*.^25^. They reported that CAV-1 overexpression in THP-1 monocytes inhibits transmigration toward MCP-1, however, CAV-1 knockdown did not have any effect on transmigration in their model. Although the exact reason for this discrepancy remains unclear, there are several possible reasons that may have caused it; e.g. the difference in type of cells (THP-1 or primary monocyte), the proportion of CAV-1-reduced cells induced by transfection, and other methodological differences (used cell numbers, incubation time, and procedures). Elevated MCP-1 expression level was found in psoriasis skin^13,26^. CCR2, a receptor for MCP-1, is also overexpressed in monocytes and macrophages of patients with psoriasis^27^. The interaction of MCP-1 and CCR2 is supposed to be crucial in trafficking monocytes into psoriatic skin lesions^28^. Our observation implies that CAV-1 reduction on monocytes may be responsible for augmentation or maintenance of this system. Monocytes infiltrate into the skin while differentiating into a part of macrophages in peripheral tissues. Macrophages are central regulators of immune response under various pathologies, and their differentiation and behaviour are largely thought to be regulated by soluble factors in the microenvironment. How CAV-1 reduction in psoriatic monocytes affects macrophage differentiation and its function remains unknown. In this regard, Pavlides *et al*. reported that bone marrow-derived macrophages from CAV-1 deficient mice are more susceptible to apoptosis and more prone to induce inflammation^29^. This finding may suggest that diminished CAV-1 monocytes may be a source of inflammatory macrophages in psoriasis. On the other hand, a recent study showed that CAV-1 deficiency in monocytes may inhibit macrophage differentiation^25^. Because monocyte-derived macrophages serve various function, further studies are required to clarify the role of CAV-1 on monocytes/macrophages in the circumstance of psoriasis milieu. In particular, CAV-1 deficiency on monocytes seems to be implicated on atherosclerosis; the association of CAV-1 impairment and psoriasis comorbidity may be a prospective study with great interest.

Given the association between reduced CAV-1 expression and psoriatic inflammation in human monocytes, we investigated the effect of manipulating CAV-1 function using CSD peptides *in vivo* in mice experiments. We have previously administrated CSD peptide intradermally at the same area of the IMQ topical site to determine the direct response of CSD to lesional keratinocytes^3^. In the present study, we focused on CAV-1 deficiency on leukocytes/monocytes and their contribution to psoriatic inflammation, therefore CSD was systemically administrated by intraperitoneal injection to avoid direct contact with lesional keratinocytes. A significant suppression of CAV-1 level in PBMCs was validated in IMQ-treated mice, and functional recovery of CAV-1 by intraperitoneal administration of CSD peptides not only improved the cutaneous phenotype, but also reduced the number of infiltrated dermal macrophages. However, our study shows the possibility that decreased infiltration of dermal macrophage may not be due solely to the effect of CSD peptide on monocytes but also the secondary effect of skin improvement. Moreover, we believe that CAV-1 deficiency in both keratinocytes and monocytes, at least in part, participates and coordinates in the formation and maintenance of psoriatic skin lesions, but it is unclear which cells are dominant contributors to psoriatic skin formation. This is a limitation of our study and further experiments are desired using transgenic mice in which CAV-1 was knocked out specifically on the skin or monocytes in order to clarify the direct contribution of each.

Previous reports showed that CSD peptides do not affect CAV-1 expression, and CSD peptides themselves are not detected as they are only a short functional domain of CAV-1 and only mimic CAV-1 function^30,31^. However, we observed that CAV-1 expression tended to be reversed by administering CSD peptides in PBMCs, but no such tendency was found in dermal macrophages. Although the reason for this variation on CAV-1 level caused by CSD treatment is unclear, several reasonable explanations exist regarding our observation. First, treatment with CSD peptides ameliorated psoriasis inflammation, which was a trigger for CAV-1 downregulation. Thus, CAV-1 level was reversed as a consequence of elimination of the cause for CAV-1 inhibition in the blood. Second, CAV-1-reduced monocytes had higher migration activity. Hence, the absence of difference is reasonable in CAV-1 level on dermal macrophages after CSD treatment, as those cells were already migrated because of reduced CAV-1 level. Third, a study reported that CAV-1 expression is elevated after monocyte-to-macrophage differentiation in both immortalized and primary cells^25^. CAV-1 level was also possibly altered during differentiation in our experiments.

In summary, our study suggested that CAV-1 reduction in circulating monocytes of patients with psoriasis may play a role in the pathogenesis of psoriasis by enhancing cytokine production and promoting monocyte infiltration into the skin. Furthermore, recovery of CAV-1 function by systemic administration of CSD peptides improved skin phenotype through adjustment of skin infiltration of macrophages, demonstrating that CAV-1 may be a therapeutic target.

## Materials and Methods

### Patients

Twenty patients who were diagnosed with psoriasis vulgaris were included in the study. Blood and skin samples were obtained from patients with psoriasis who had no current immunosuppressive medications and no history of biologics. Serial samples were collected before treatment and after clinical improvement by biologics in some patients. Clinical features of patients are shown in Supplementary Tables S1 and S2. Written informed consent was obtained from all patients in accordance with the Declaration of Helsinki. All methods were carried out in accordance with the relevant guidelines and regulations. The study was approved by the Institutional Review Board of Yokohama City University (Approval No: B120705021).

### Cell isolation

Peripheral blood samples were collected, and PBMCs and PMNs were isolated by density centrifugation at 700 *g* for 30 min using Histopaque^®^ 1119 and 1077 (Sigma-Aldrich, St Louis, MO). Separated cell layers were suspended in RPMI 1640 (Wako Pure Chemical Industries, Osaka, Japan). Human primary monocytes were purified from PBMCs using the Monocyte Isolation Kit II (Miltenyi Biotec, Bergisch Gladbach, Germany). The purity of isolated monocytes was more than 85% and validated through the expression of CD14 by flow cytometry (Supplementary Fig. S2). Cell suspensions were prepared for immunoblotting and PCR analyses as appropriate.

### Semi-quantitative and quantitative polymerase chain reaction (qPCR)

Total RNA was extracted using TRIzol^®^ and the Illustra^TM^ RNAspin Mini RNA Isolation Kit (GE Healthcare, Uppsala, Sweden), and qPCR was performed in duplicate using TaqMan^®^ gene expression assays or the Thunderbird^®^ SYBR^®^ qPCR Mix (Toyobo, Osaka, Japan) as previously described^3^. Gene expression levels were normalized to *β-actin* or *GAPDH* and compared using the 2^*−ΔΔCt*^ method. TaqMan^®^ probes for human *CAV-1*, *TNF-α*, *IL-1β*, *IL-17A*, *IL-6*, *IL-10*, and *β-actin*, mouse *β-actin*, and *CAV-1* were obtained from Applied Biosystems (Carlsbad, CA); the assay identification numbers are shown in Supplementary Table S3. The primer sets used for semi-quantitative PCR and SYBR qPCR are shown in Supplementary Table S4.

### Immunoblotting

Samples were prepared using radioimmunoprecipitation assay buffer containing a protease inhibitor cocktail as previously described^32^. Equal quantities of protein were analysed by immunoblotting using one of the following antibodies: CAV-1 (Santa Cruz Biotechnology, Santa Cruz, CA) and GAPDH (Cell Signaling). Signals were detected using horseradish peroxidase-conjugated secondary antibody and ECL^TM^ Prime Western Blotting Detection Reagent (GE Healthcare).

### Immunocytostaining

PBMCs were collected by cytospin and stained using the indicated primary antibodies. Cells were fixed with 2% paraformaldehyde and then permeabilized with 0.1% Triton X-100. Cells were incubated with Alexa Flour^®^ 647-conjugated anti-CD3, CD14, or CD19 (BioLegend, San Diego, CA), and anti-CAV-1 (Santa Cruz Biotechnology), followed by Alexa Flour^®^ 488-conjugated secondary IgG (Invitrogen, Grand Island, NY). Appropriate isotype IgG was used as a control. ProLong Gold antifade reagent with 4,6-diamidino-2-phenylindole (DAPI; Life Technology, Carlsbad, CA) was used for nuclear identification and mounting. The intensity of CAV-1 staining was quantified in randomly chosen CD3^+^ T cells, CD19^+^ B cells, and CD14^+^ monocytes. The data were obtained from 20 cells from each three patients and healthy subjects. Images were taken and analysed using an Olympus Fluoview 1000 microscope (Olympus America, Melville, NY) and fixed camera settings.

### CAV-1 silencing

Purified monocytes (3 × 10^5^) were seeded on a 24-well multidish (Thermo Fisher Scientific, Rochester, NY) in RPMI-1640 supplemented with 10% foetal bovine serum. CAV-1-specific small-interfering RNA (Stealth RNAi) and control RNAi were purchased from Invitrogen. For transfection, Viromer^®^ Green (Lipocalyx GmbH, Halle (Saale), Germany) was used in accordance with the manufacturer’s instructions. A mixture of 50 pM of each RNAi and Viromer^®^ was added to cells, and cells were cultured for 24 h. Serum was starved at least 6 h before further stimulation. CAV-1-silenced or controlled monocytes were cultured supplemented with 50 ng/mL LPS (Sigma-Aldrich) and harvested 4 h after stimulation. The culture supernatants were collected by centrifugation and aliquoted into small sterile tubes. All the samples were stored at −80 °C until assayed for cytokines.

### Enzyme-linked immunosorbent assay

The levels of IL-1 and IL-6 of supernatant samples were measured on duplicate samples using Quantikine^®^ ELISA kits (R&D Systems Europe, Abingdon, U.K.) according to the manufacturer’s instructions.

### Monocyte migration assay

Monocyte chemoattractant activity was assessed using 24-well TransWell^®^ plates with 5-μm pore filters (Corning Incorporated, Corning, NY). CAV-1-silenced monocytes (5 × 10^3^) or controlled monocytes (5 × 10^3^) were placed in the upper compartment of each chamber. RPMI-1640 supplemented with 1% FBS in either the presence or absence of 100 ng/ml MCP-1 (Thermo Fisher Scientific) was added into the lower compartment. Cells were allowed to migrate through the filter for 90 min at 37 °C and then harvested from both compartments and manually counted with a haemocytometer. Percentage of migrated cell was calculated as ratio of the cell count of the lower compartment to the total cell count of the upper and lower compartments.

### Murine model of psoriasis-like skin inflammation

For *in vivo* experiments, 6-week-old C57BL/6 J female mice (Japan SLC, Shizuoka, Japan) were used. IMQ was used to induce skin inflammation as previously described^3^. A daily dose of 62.5 mg of 5% IMQ cream (Beselna^®^ cream; Mochida Pharmaceutical, Tokyo, Japan) was applied to the back skin of mice for 10 consecutive days. Vaseline was used as control. On another psoriasis-like murine model, IL-23 was used to induce skin inflammation as previously described^33^. Twenty millilitres of PBS containing 500 ng recombinant mouse IL-23 (BioLegend, San Diego, CA) or the same amount of PBS alone was intradermally administered into ears every other day for 10 days. All animal experiments were performed after approval by Yokohama City University Institutional Animal Care and Use Committee (Approval No: F-A-16–087), and in accordance with relevant guidelines and regulations.

### CSD peptide treatment

Peptides corresponding to the CSD (DGIWKASFTTFTVTKYWFYR) and a scrambled control peptide (Ctr: WGIDKAFFTTSTVTYKWFRY) fused to the antennapedia internalization sequence (RQIKIWFQN RRMKWKK) were synthesized and dissolved in dimethyl sulfoxide. For injection, peptides were diluted in PBS, and 100 μl of 0.1 mM solution of the CSD or Ctr peptide was injected into mice intraperitoneally from day 1 (the first day of IMQ or IL-23 treatment) to day 10.

### Harvesting blood and skin samples in murine model

Peripheral blood was collected by cardiac puncture in the presence of heparin at day 11. Blood was subjected to density gradient separation on Lympholyte^®^ Mammal (Cedarlane, Burlington, Ontario, Canada) and centrifuged at 800 *g* for 20 min. PBMC layer was collected, and cell suspensions were prepared for PCR analyses as appropriate. Mice back skin where Vaseline or IMQ cream was applied was harvested at day 11 and homogenized with Micro Smash^TM^ (TOMY SEIKO, Tokyo, Japan) for PCR analyses as appropriate.

### Measurement of the clinical skin scores, epidermal thickness, and dermal macrophages in mice

The clinical skin score of IMQ-induced psoriasis inflammation mice was determined from day 1 (the first day of treatment) to day 11 using the modified psoriasis severity index score as previously described^3^. In IL-23-induced psoriasis-like mice, ear thickness was measured before injection on day 1 and thereafter for 10 days before injections. Ear measurements were made at the centre of the ears using an Adjustable Measuring Force Digimatic Micrometer (Mitutoyo, Kawasaki, Japan). All measurements were performed with blinding. Sections from paraffin-embedded mouse skin samples were stained with haematoxylin and eosin. The thickness of the epidermis was measured from the stratum basale to the stratum granulosum as previously described^3^. A number of infiltrated cells were also counted from 5 random fields of view of 10,000 μm^2^ for each mouse. Immunohistochemical analysis of CAV-1 was performed as previously described^3^. Briefly, sections were incubated with antibodies against CAV-1 (Santa Cruz Biotechnology) or isotype controls. The antigen was detected using EnVision Dual Link System-HRP or LSAB2 system-HRP, and AEC^+^ high sensitivity substrate chromogen was applied (Dako, Carpinteria, CA). Dermal macrophages were validated by immunofluorescent staining. Frozen cryosections of skin were stained with anti-F4/80 (Bio-Rad, Hercules, CA) and anti-CAV-1, followed by Alexa Flour^®^ 633- and 488-conjugated secondary IgG, respectively. The intensity of CAV-1 staining in macrophages was quantified at the average of 10 infiltrated F4/80^+^ cells of 10 random fields for each mouse.

### Statistical analysis

Statistical comparisons were performed using Mann-Whitney U test, unpaired Student’s t-test, Wilcoxon test, or one-way analysis of variance (post-hoc Tukey or Dunnett) as indicated. All tests were carried out using GraphPad Prism version 7.0 software (GraphPad Software, San Diego, CA). A P value < 0.05 was considered significant.

## Electronic supplementary material

Supplementary Information

## References

[1] Griffiths CE, Barker JN (2007). Pathogenesis and clinical features of psoriasis. Lancet.

[2] Lowes MA, Bowcock AM, Krueger JG (2007). Pathogenesis and therapy of psoriasis. Nature.

[3] Yamaguchi Y, Watanabe Y, Watanabe T, Komitsu N, Aihara M (2015). Decreased Expression of Caveolin-1 Contributes to the Pathogenesis of Psoriasiform Dermatitis in Mice. J Invest Dermatol.

[4] Kiss AL, Turi A, Mullner N, Timar J (2000). Caveolin isoforms in resident and elicited rat peritoneal macrophages. Eur J Cell Biol.

[5] Yan SR, Fumagalli L, Berton G (1996). Activation of SRC family kinases in human neutrophils. Evidence that p58C-FGR and p53/56LYN redistributed to a Triton X-100-insoluble cytoskeletal fraction, also enriched in the caveolar protein caveolin, display an enhanced kinase activity. FEBS letters.

[6] Shin JS, Gao Z, Abraham SN (2000). Involvement of cellular caveolae in bacterial entry into mast cells. Science.

[7] Harris J, Werling D, Hope JC, Taylor G, Howard CJ (2002). Caveolae and caveolin in immune cells: distribution and functions. Trends in immunology.

[8] Wang XM, Kim HP, Song R, Choi AM (2006). Caveolin-1 confers antiinflammatory effects in murine macrophages via the MKK3/p38 MAPK pathway. Am J Respir Cell Mol Biol.

[9] Tourkina E (2010). Caveolin-1 regulates leucocyte behaviour in fibrotic lung disease. Ann Rheum Dis.

[10] Schonle A (2016). Caveolin-1 regulates TCR signal strength and regulatory T-cell differentiation into alloreactive T cells. Blood.

[11] Engel D (2011). Caveolin-1 deficiency decreases atherosclerosis by hampering leukocyte influx into the arterial wall and generating a regulatory T-cell response. FASEB J.

[12] Zhu T, Meng Q, Ji J, Zhang L, Lou X (2017). TLR4 and Caveolin-1 in Monocytes Are Associated With Inflammatory Conditions in Diabetic Neuropathy. Clin Transl Sci.

[13] Deleuran M (1996). Localization of monocyte chemotactic and activating factor (MCAF/MCP-1) in psoriasis. J Dermatol Sci.

[14] Fuentes-Duculan J (2010). A subpopulation of CD163-positive macrophages is classically activated in psoriasis. J Invest Dermatol.

[15] Bucci M (2000). *In vivo* delivery of the caveolin-1 scaffolding domain inhibits nitric oxide synthesis and reduces inflammation. Nat Med.

[16] Del Galdo F (2008). Decreased expression of caveolin 1 in patients with systemic sclerosis: crucial role in the pathogenesis of tissue fibrosis. Arthritis Rheum.

[17] Takahashi H, Tsuji H, Hashimoto Y, Ishida-Yamamoto A, Iizuka H (2010). Serum cytokines and growth factor levels in Japanese patients with psoriasis. Clin Exp Dermatol.

[18] Suarez-Farinas M (2012). Expanding the psoriasis disease profile: interrogation of the skin and serum of patients with moderate-to-severe psoriasis. J Invest Dermatol.

[19] Kyriakou A, Patsatsi A, Vyzantiadis TA, Sotiriadis D (2014). Serum levels of TNF-alpha, IL-12/23p40, and IL-17 in plaque psoriasis and their correlation with disease severity. J Immunol Res.

[20] Okubo Y, Koga M (1998). Peripheral blood monocytes in psoriatic patients overproduce cytokines. J Dermatol Sci.

[21] Mizutani H, Ohmoto Y, Mizutani T, Murata M, Shimizu M (1997). Role of increased production of monocytes TNF-alpha, IL-1beta and IL-6 in psoriasis: relation to focal infection, disease activity and responses to treatments. J Dermatol Sci.

[22] Mizutani H, Ohmoto Y, Tanaka H, Shimizu M (1997). Psoriatic monocytes respond sensitively to lipopolysaccharide but with limited inflammatory cytokine production. Archives of dermatological research.

[23] Yamanaka K (2014). Biologic therapy improves psoriasis by decreasing the activity of monocytes and neutrophils. J Dermatol.

[24] Haines P, Samuel GH, Cohen H, Trojanowska M, Bujor AM (2011). Caveolin-1 is a negative regulator of MMP-1 gene expression in human dermal fibroblasts via inhibition of Erk1/2/Ets1 signaling pathway. J Dermatol Sci.

[25] Yi F XLM (2012). Caveolin-1 Plays a Critical Role in the Differentiation of Monocytes into Macrophages. Arterioscler Thromb Vasc Biol..

[26] Gillitzer R (1993). MCP-1 mRNA expression in basal keratinocytes of psoriatic lesions. J Invest Dermatol.

[27] Vestergaard C, Just H, Baumgartner Nielsen J, Thestrup-Pedersen K, Deleuran M (2004). Expression of CCR2 on monocytes and macrophages in chronically inflamed skin in atopic dermatitis and psoriasis. Acta dermato-venereologica.

[28] Lembo S (2014). MCP-1 in psoriatic patients: effect of biological therapy. J Dermatolog Treat.

[29] Pavlides S (2014). Caveolin-1 regulates the anti-atherogenic properties of macrophages. Cell Tissue Res.

[30] Weng P (2017). Caveolin-1 scaffolding domain peptides enhance anti-inflammatory effect of heme oxygenase-1 through interrupting its interact with caveolin-1. Oncotarget.

[31] Tourkina E (2008). Antifibrotic properties of caveolin-1 scaffolding domain *in vitro* and *in vivo*. Am J Physiol Lung Cell Mol Physiol.

[32] Yamaguchi Y, Yasuoka H, Stolz DB, Feghali-Bostwick CA (2011). Decreased caveolin-1 levels contribute to fibrosis and deposition of extracellular IGFBP-5. J Cell Mol Med.

[33] Mabuchi T (2013). CCR6 is required for epidermal trafficking of gammadelta-T cells in an IL-23-induced model of psoriasiform dermatitis. J Invest Dermatol.

