# Supplementary material for: Downregulated Caveolin-1 expression in circulating monocytes may contribute to the pathogenesis of psoriasis
Source: Sci Rep. 2019 Jan 15;9:125. doi: 10.1038/s41598-018-36767-5 (PMC6333821; doi:10.1038/s41598-018-36767-5)
Supplement: Supplementary file 1 — Supplementary Information [file 41598_2018_36767_MOESM1_ESM.pdf]

Supplementary information

Downregulated Caveolin-1 expression in circulating monocytes may contribute to the pathogenesis of psoriasis

Naoko Takamura, Yukie Yamaguchi, Yuko Watanabe, Miho Asami, Noriko Komitsu, Michiko Aihara

Department of Environmental Immuno-Dermatology, Yokohama City University Graduate School of Medicine, Yokohama, Japan

***Corresponding author*:**

Yukie Yamaguchi MD, PhD

Department of Environmental Immuno-Dermatology, Yokohama City University Graduate School of Medicine

3-9 Fukuura, Kanazawa-ku, Yokohama, Kanagawa, 236-0004, Japan

Tel: +81 45 787 2675

Fax: +81 45 786 0243

E-mail: yui1783@yokohama-cu.ac.jp

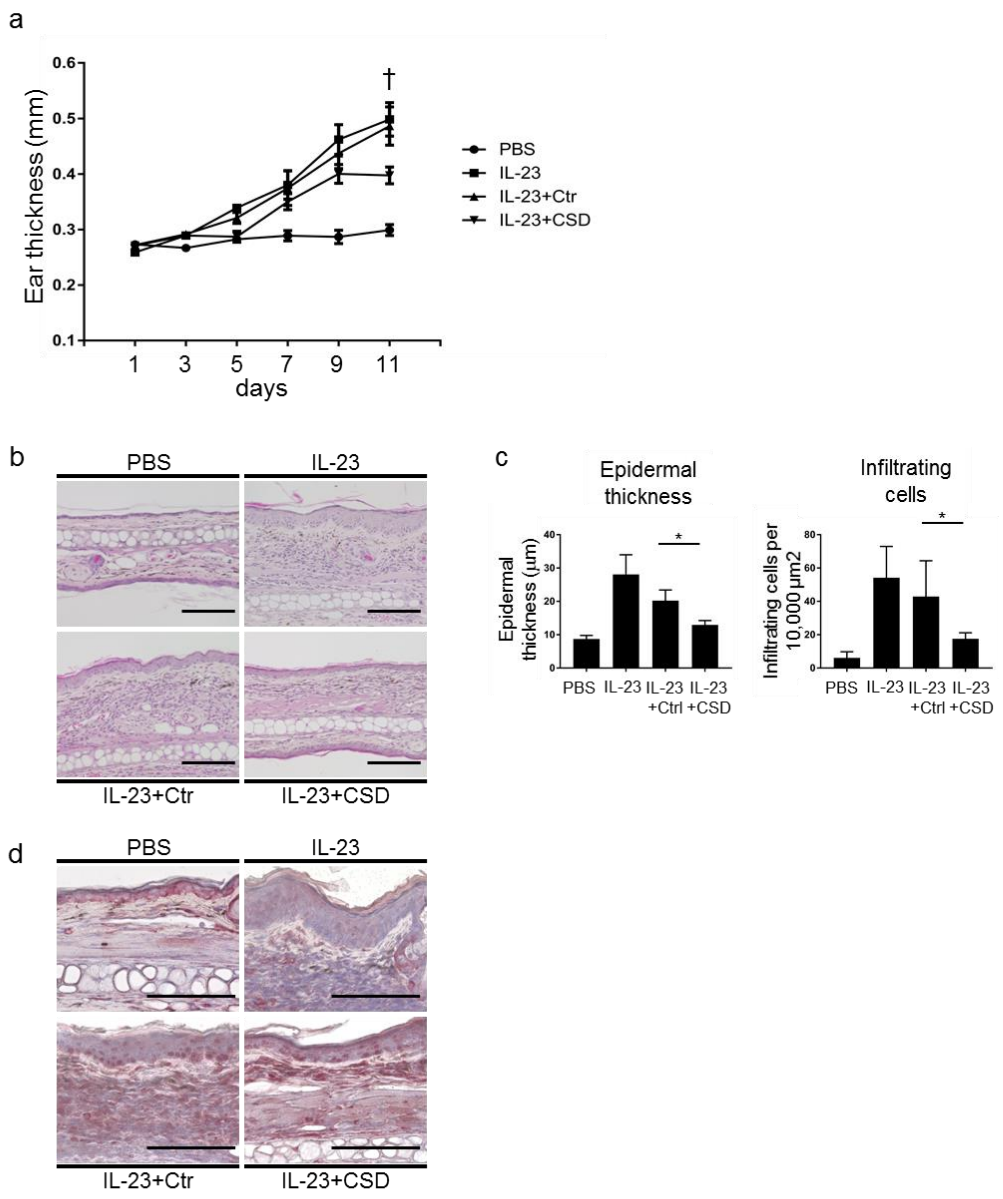

Supplementary Figure S1. Phenotype of IL-23-induced murine model of psoriasis-like skin inflammation and suppressed inflammation by systemic administration of CSD peptide. (a) Mice were treated with IL-23 alone (IL-23), IL-23 and the CAV-1 scaffolding domain (CSD) peptide (IL-23 + CSD), IL-23 and the control peptide (IL-23 + Ctr), or PBS every other day for 10 days. Ear thickness in each group: PBS (circles); IL-23 alone (squares); IL-23 + CSD (inverted triangles); and IL-23 + Ctr (triangles). Graphs indicate the mean ± SD of each group (n = 5). One-way analysis of variance (post hoc Tukey) revealed †P < 0.01 (IL-23 vs. IL-23 + CSD). (b) Representative haematoxylin and eosin staining of mice skin. Scale bar = 100 µm. (c) The epidermal thickness and the number of infiltrating cells were analysed. Graphs indicate the mean ± SD of each group (n = 5). One-way analysis of variance (post hoc Tukey) yielded *P < 0.05 (IL-23 + Ctr vs. IL-23 + CSD). (d) Immunohistochemical analysis of CAV-1. Scale bar = 100 µm.

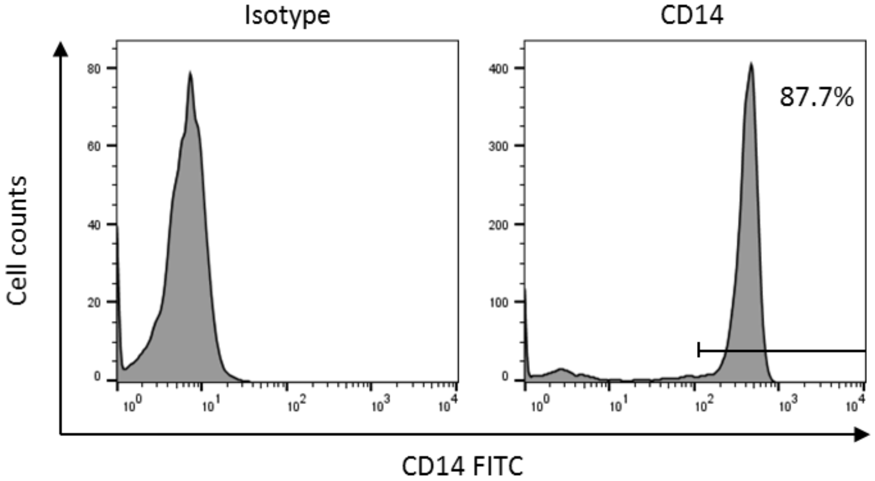

Supplementary Figure S2. Analysis of the purity of isolated monocytes from PBMCs by staining surface CD14 markers using flow cytometry. Histograms show the expression of CD14+ cells. One of representative experiment is shown.

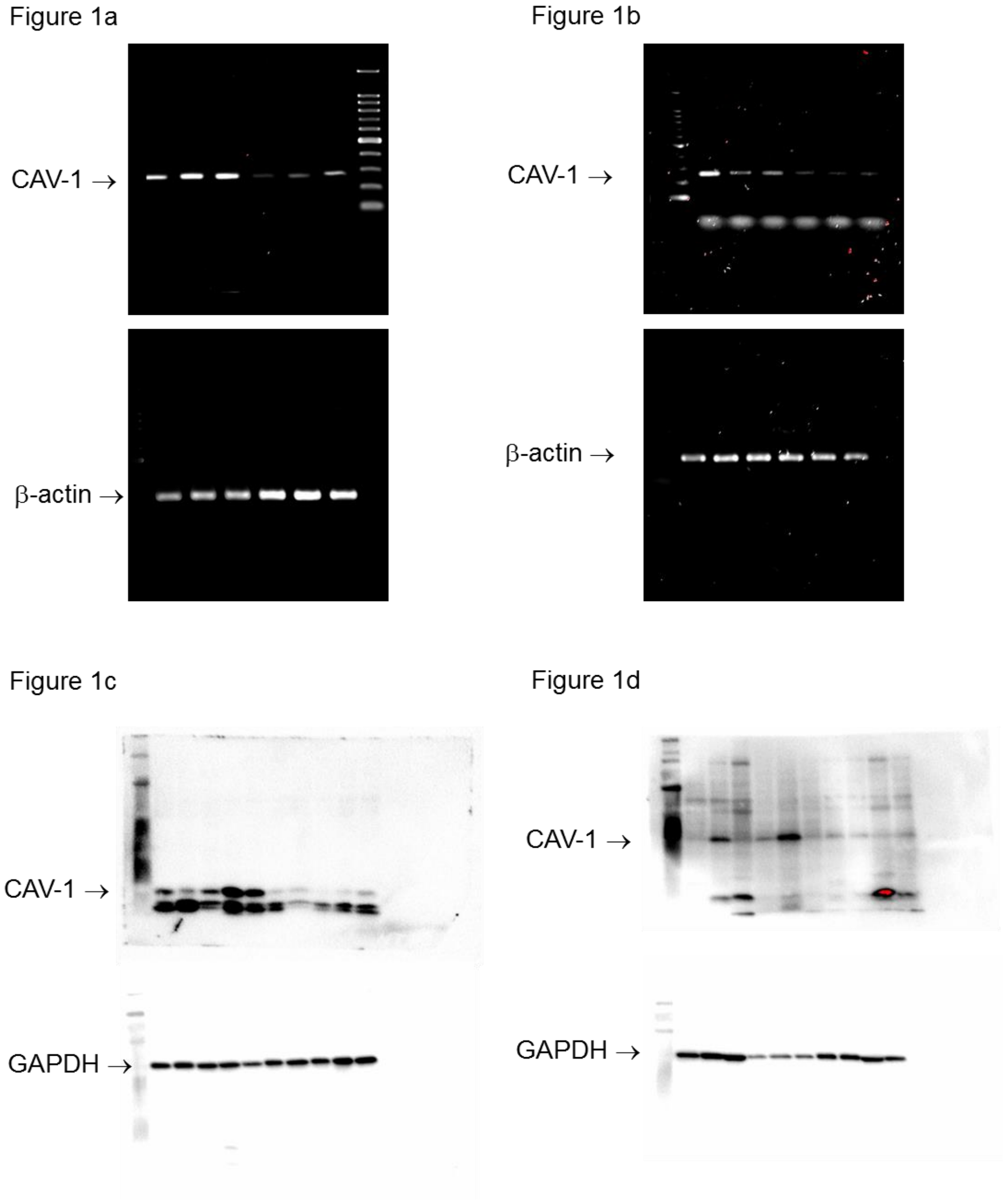

Supplementary Figure S3. Decreased CAV-1 expression in PBMCs and PMNs from patients with psoriasis. CAV-1 expression in PBMCs (Figure 1a, c) and PMNs (Figure 1b, d) of patients with psoriasis (PSO) and healthy subjects (HC) was evaluated using semi-quantitative and qPCR (Figure 1a, b), and immunoblotting (Figure 1c, d). Full scan images of immunoblots for main figures.

Figure 2c

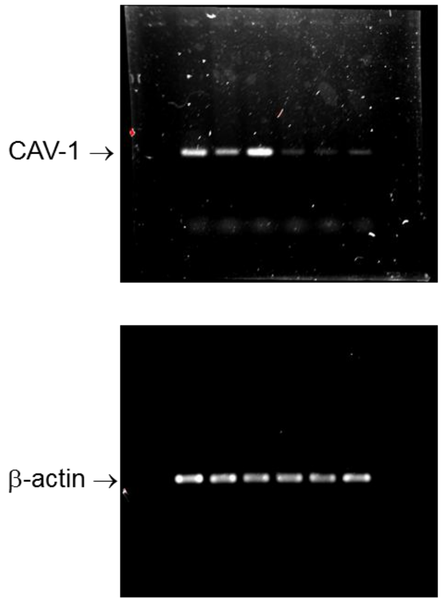

Figure 2d

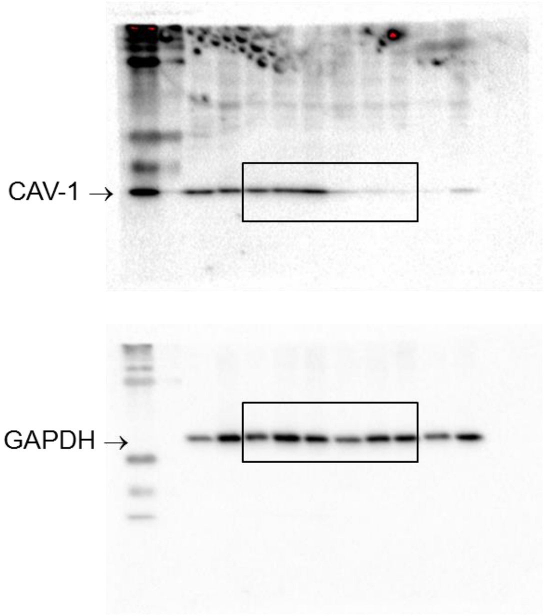

Supplementary Figure S4. Diminished CAV-1 expression in circulating monocytes of patient with psoriasis. CAV-1 levels in purified monocytes were evaluated using semi-quantitative and qPCR (Figure 2c), and immunoblotting (Figure 2d). Full scan images of immunoblots for main figures.

Figure 5g

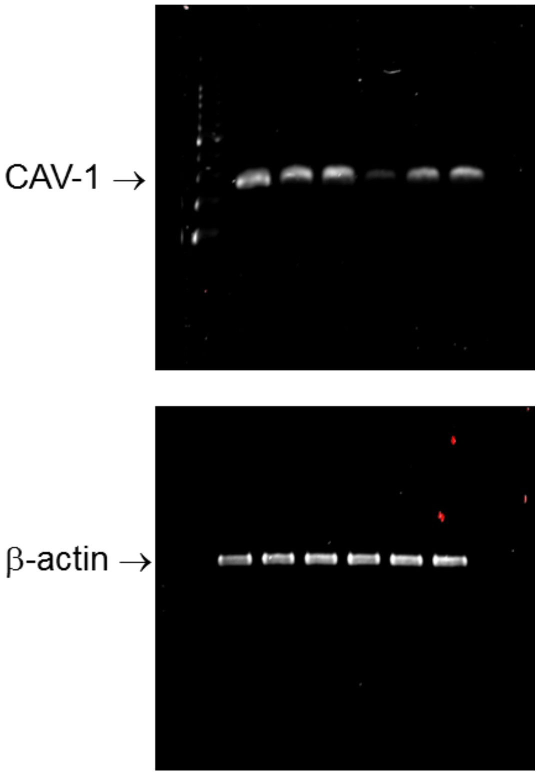

Supplementary Figure S5. Phenotype of IMQ-induced murine model of psoriasis-like skin inflammation and suppressed inflammation by systemic administration of CSD peptide. CAV-1 expression in PBMCs in each group was determined using semi-quantitative qPCR (Figure 5g). Full scan images of immunoblots for main figures.

Supplementary Table S1: Clinical Features of Psoriasis Patients and control subjects.

Clinical Features of Psoriasis Patients and control subjects

	**Psoriasis (n=20)**		**Healthy controls (n=20)**
Gender	M 16, F 4		M 11, F 9
Age: Mean ± SD (range), yr	52.9 ± 13.3 (23-72)		46.4 ± 14 (24-73)
Body Mass Index: Mean ± SD (range)	25.6 ± 5.3 (19.2-41)		-
PASIscore: Mean ± SD (range)	16.5 ± 8 (7-35.2)		-
Disease duration: Mean ± SD (range), yr	17.2 ± 14.5 (1-44)		-
Medications (current)	No medication	5	
	Topical therapy	14	
	Phototherapy	5	
	Etretinate	2	

Supplementary Table S2: Clinical Features of Psoriasis Patients treated with biologics.

Clinical Features of Psoriasis Patients treated with biologics

	**Psoriasis patients treated with biologics (n = 12)**	
Gender	M 7, F 5	
Age: Mean ± SD (range), yr	59.8 ± 11.2 (35-72)	
PASIscore before treatment: Mean ± SD (range)	15.2 ± 9.1 (0-35.2)	
PASIscore after treatment: Mean ± SD (range)	4.6 ± 7.7 (0-30)	
Type of psoriasis	Psoriasis vulgaris	9
	Psoriatic arthritis	2
	Generalized pustular psoriasis	1
Medications (before biologics)	No medication	1
	Topical therapy	6
	Phototherapy	1
	Cyclosporine	4
	Methotrexate	1
Medications (current)	TNF-α inhibitor	7
	IL-17A inhibitor	2
	IL-23 inhibitor	3

Supplementary Table S3: TaqMan® probes assay identification numbers.

TaqMan® probes assay identification numbers

TaqMan® probes	Assay identification number
Human Cav-1	Hs00971716_m1,
Human TNF-α	Hs00174128_m1
Human IL-1β	Hs00174097_m1
Human IL-17A	Hs00174383_m1
Human IL-6	Hs00985639_m1
Human IL-10	Hs00961622_m1

Supplementary Table S4: The sequences of primers used for semi-quantitative PCR and SYBR qPCR.

Sequences of primers

Gene	Primer sequence (5→3)		Tm	Cycle
	Forward	Reverse		
Human Cav-1	GACTTTGAAGATGTGATTGC	AGATGGAATAGACACGGCTG	56	35
Human β-actin	CTACAATGAGCTGCGTGTGGC	CAGGTCCAGACGCAGGATGGC	60	28
Mouse β-actin	ATGTTTGAGACCTTCAACAC	CACGTCACACTTCATGATGG	58	28
mouse Cav-1	CCATCCGGGAACAGGGCAACA	GCCGAAGATCGTAGACAACAA	53	38

Gene	SYBR® qPCR Primer sequence (5→3)	
	Forward	Reverse
Mouse Cav-1	CGTGGTCAAGATTGACTTTGAA	ACTGTGTGTCCCTTCTGGTTCT
Mouse TNF-α	TATGGCCCAGACCCTCACACTC	CACTTGGTGGTTTGCTACGACGT
Mouse IL-23p19	ATAATGTGCCCCGTATCCAG	AAGCAGAACTGGCTGTTGTC
Mouse IL-17A	TCCAGAAGGCCCTCAGACTA	CTTCATTGCGGTGGAGAGTC
Mouse IFN-α	CCTGAGARAGAAGAAACACAGCC	GGCTCTCCAGAYTTCTGCTCTG
